# *ARHGAP10*, which encodes Rho GTPase-activating protein 10, is a novel gene for schizophrenia risk

**DOI:** 10.1038/s41398-020-00917-z

**Published:** 2020-07-22

**Authors:** Mariko Sekiguchi, Akira Sobue, Itaru Kushima, Chenyao Wang, Yuko Arioka, Hidekazu Kato, Akiko Kodama, Hisako Kubo, Norimichi Ito, Masahito Sawahata, Kazuhiro Hada, Ryosuke Ikeda, Mio Shinno, Chikara Mizukoshi, Keita Tsujimura, Akira Yoshimi, Kanako Ishizuka, Yuto Takasaki, Hiroki Kimura, Jingrui Xing, Yanjie Yu, Maeri Yamamoto, Takashi Okada, Emiko Shishido, Toshiya Inada, Masahiro Nakatochi, Tetsuya Takano, Keisuke Kuroda, Mutsuki Amano, Branko Aleksic, Takashi Yamomoto, Tetsushi Sakuma, Tomomi Aida, Kohichi Tanaka, Ryota Hashimoto, Makoto Arai, Masashi Ikeda, Nakao Iwata, Teppei Shimamura, Taku Nagai, Toshitaka Nabeshima, Kozo Kaibuchi, Kiyofumi Yamada, Daisuke Mori, Norio Ozaki

**Affiliations:** 1 Department of Psychiatry, Nagoya University Graduate School of Medicine, Nagoya, Aichi Japan; 2 Department of Pharmacology, Nagoya University Graduate School of Medicine, Nagoya, Aichi Japan; 3 Department of Neuropsychopharmacology and Hospital Pharmacy, Nagoya University, Graduate School of Medicine, Nagoya, Aichi Japan; 4 Medical Genomics Center, Nagoya University Hospital, Nagoya, Aichi Japan; 5 Center for Advanced Medicine and Clinical Research, Nagoya University Hospital, Nagoya, Aichi Japan; 6 Division of Data Science, Department of Nursing, Nagoya University Graduate School of Medicine, Nagoya, Aichi Japan; 7 Division of Integrated Sciences for Life, Graduate School of Integrated Sciences for Life, Hiroshima University, Hiroshima, Japan; 8 Laboratory of Molecular Neuroscience, Medical Research Institute, Tokyo Medical and Dental University, Tokyo, Japan; 9 Department of Pathology of Mental Diseases, National Institute of Mental Health, National Center of Neurology and Psychiatry, Kodaira, Tokyo, Japan; 10 Molecular Research Center for Children’s Mental Development, United Graduate School of Child Development, Osaka University, Suita, Osaka, Japan; 11 Department of Psychiatry, Osaka University Graduate School of Medicine, Suita, Osaka, Japan; 12 Department of Psychiatry and Behavioral Sciences, Tokyo Metropolitan Institute of Medical Science, Tokyo, Japan; 13 Department of Psychiatry, Fujita Health University School of Medicine, Toyoake, Japan; 14 Division of Systems Biology, Nagoya University Graduate School of Medicine, Nagoya, Aichi Japan; 15 Advanced Diagnostic System Research Laboratory Fujita Health University, Graduate School of Health Sciences & Aino University, Toyoake, Aichi Japan; 16 Brain and Mind Research Center, Nagoya University, Nagoya, Aichi Japan

**Keywords:** Molecular neuroscience, Clinical genetics

## Abstract

Schizophrenia (SCZ) is known to be a heritable disorder; however, its multifactorial nature has significantly hampered attempts to establish its pathogenesis. Therefore, in this study, we performed genome-wide copy-number variation (CNV) analysis of 2940 patients with SCZ and 2402 control subjects and identified a statistically significant association between SCZ and exonic CNVs in the *ARHGAP10* gene. *ARHGAP10* encodes a member of the RhoGAP superfamily of proteins that is involved in small GTPase signaling. This signaling pathway is one of the SCZ-associated pathways and may contribute to neural development and function. However, the *ARHGAP10* gene is often confused with *ARHGAP21*, thus, the significance of *ARHGAP10* in the molecular pathology of SCZ, including the expression profile of the ARHGAP10 protein, remains poorly understood. To address this issue, we focused on one patient identified to have both an exonic deletion and a missense variant (p.S490P) in *ARHGAP10*. The missense variant was found to be located in the RhoGAP domain and was determined to be relevant to the association between *ARHGAP10* and the active form of RhoA. We evaluated ARHGAP10 protein expression in the brains of reporter mice and generated a mouse model to mimic the patient case. The model exhibited abnormal emotional behaviors, along with reduced spine density in the medial prefrontal cortex (mPFC). In addition, primary cultured neurons prepared from the mouse model brain exhibited immature neurites in vitro. Furthermore, we established induced pluripotent stem cells (iPSCs) from this patient, and differentiated them into tyrosine hydroxylase (TH)-positive neurons in order to analyze their morphological phenotypes. TH-positive neurons differentiated from the patient-derived iPSCs exhibited severe defects in both neurite length and branch number; these defects were restored by the addition of the Rho-kinase inhibitor, Y-27632. Collectively, our findings suggest that rare *ARHGAP10* variants may be genetically and biologically associated with SCZ and indicate that Rho signaling represents a promising drug discovery target for SCZ treatment.

## Introduction

Schizophrenia (SCZ) is a severe psychiatric disorder characterized by hallucinations, delusions, and cognitive deficits, with a lifetime risk of ~1% and a heritability of up to 80%^1^. To further investigate the genetic basis of this disorder, various genome-wide studies have been performed and have revealed an important role for rare (<1%) copy-number variants (CNVs) in SCZ^2–4^. Indeed, these studies have identified rare CNVs at specific loci as strong risk factors for SCZ. While many of the identified CNVs are large (>1 Mb) recurrent CNVs encompassing many genes (e.g., deletions at 22q11.2 and 3q29), small deletions in specific genes (e.g., *NRXN1*) have also been implicated in this disorder. Furthermore, gene set analyses of the genes affected by CNVs have implicated multiple biological pathways in the pathogenesis of SCZ. One of these SCZ-associated pathways is small GTPase signaling^2,3,5^, which is activated by guanine nucleotide exchange factors (GEFs) and inactivated by GTPase-activating proteins. The Rho family of small GTPases is involved in multiple aspects of neuronal development through the regulation of cytoskeletal rearrangements, cell motility, cell polarity, and axon guidance^6–8^. In addition, genes encoding RhoGAPs, including *ARHGAP33*, *OPHN1*, and *SRGAP3*, have been implicated in psychiatric and neurodevelopmental disorders^9–11^. For example, a dysfunction in *OPHN1* has been shown to compromise spine morphogenesis, suggesting that losing *OPHN1* increases signaling through RhoA/Rho-kinases, leading to altered dendritic spine morphology^12,13^.

In this study, we found rare exonic CNVs of *ARHGAP10* in SCZ patients, along with evidence for a genetic association between *ARHGAP10* and SCZ. *ARHGAP10* is a member of the RhoGAP superfamily of proteins involved in small GTPase signaling, which has been implicated in the pathogenesis of SCZ by our work and those of others^2,3,5^. *ARHGAP10* mRNA is predominantly expressed in the brain, heart, skeletal muscle, and testis^14,15^. While *ARHGAP10* has not been previously implicated in SCZ, rare CNVs in this gene have been reported in patients with various brain disorders, including generalized seizures, intellectual disabilities, and ventriculomegaly, suggesting its clinical significance^16,17^. While the biological function and protein expression profile of ARHGAP10 have not been fully determined, it is believed that, as a RhoGAP protein, ARHGAP10 stimulates the intrinsic GTPase activity of RhoA and inactivates it^14,15^. RhoA is a member of the Rho family of GTPases and regulates actin cytoskeleton destabilization, such that RhoA activation results in both decreased dendritic growth and branching, as well as reduced dendritic spine density^18,19^. In addition, the polarized activation of RhoA/Rho-kinase in the cell body is required for minor neurite retraction and single axon formation, which are highly regulated by the Ca^2^^+^/CaMKI/GEF-H1/RhoA/Rho-kinase signaling pathway^20,21^. Rho-kinase phosphorylates and inactivates p190RhoGAP, a member of the RhoGAP family, thereby leading to sustained RhoA activation and the eventual guarantee of neuronal polarity through multiple pathways in the minor neurites^20,22^.

In this study, we hypothesized that *ARHGAP10* is associated with neuronal polarity and its genetic variants cause neurodevelopmental abnormalities related to SCZ. However, since *ARHGAP10* is often confused with *ARHGAP21*, the protein expression profile of the ARHGAP10 protein remains poorly understood. Therefore, in this study, we generated reporter mice encoding the full-length *Arhgap10*, along with three tandem repeats of V5 tags and the mCherry fluorescent gene, in order to further characterize ARHGAP10 expression.

In addition, to examine the role of *ARHGAP10* in the pathogenesis of SCZ, we focused on a SCZ patient who was revealed to carry an exonic deletion of *ARHGAP10* as well as a rare missense variant on the other *ARHGAP10* allele. The co-occurrence of such variants in the same gene has been recently suggested to be an important genetic mechanism of SCZ^23^. To further examine this issue, we generated a compound heterozygous mutant mouse of the same genotype as the patient and compared the in vitro phenotypes with those of induced pluripotent stem cells (iPSCs) derived from the patient. We subsequently found a common in vitro phenotype of less maturation in both patient iPSC-derived tyrosine hydroxylase (TH)-positive neurons and the mutant mouse neurons. In addition, behavioral analysis of the *ARHGAP10* mutant (Case #5 model) mice demonstrated a SCZ-like phenotype suggestive of a biological association between *ARHGAP10* and SCZ. Collectively, our novel findings revealed important neuronal phenotypes underlying SCZ and demonstrated that *ARHGAP10* is likely a genetic risk factor for the disease.

## Materials and methods

### Subjects

All subjects were of Japanese ancestry and two sample sets were available for the genetic analyses performed in this study. The first sample set (3053 SCZ cases and 2451 healthy controls) was used for the association analysis for exonic CNVs in *ARHGAP10*. The second sample set (3649 SCZ cases and 3620 healthy controls) was used to examine the allele frequency of the missense variant (p.S490P) of *ARHGAP10* and its genetic association with SCZ. Supplementary Fig. 1a shows the demographic data of the two sample sets. Patients were diagnosed according to the Diagnostic and Statistical Manual of Mental Disorders, Fifth Edition criteria for SCZ. Controls were selected from the general population and had no history of psychiatric disorders based upon questionnaire responses and/or self-reporting. Written informed consent was obtained from all subjects. This study was approved by the ethics committees at each center.

### CNV analysis

Genomic DNA was extracted from blood and/or saliva samples. Array comparative genomic hybridization (aCGH) was performed using two types of arrays: the NimbleGen CGH Array 720 K (Roche NimbleGen, Madison, WI) and the Agilent 400 K CGH Array (Agilent Technologies, Santa Clara, CA). For both, CNV calls were made with Nexus Copy Number software v9.0 (BioDiscovery) using the Fast Adaptive States Segmentation Technique 2 algorithm. To obtain high-confidence CNV calls, log2 ratio thresholds for the loss and gain were set at −0.6 and 0.45, respectively. The significance threshold p-value was set at 1 × 10^−3^ and at least two contiguous probes were required for CNV calls. Using these settings, the CNV detection resolution of the two types of arrays was similar. A noise-reduction algorithm for the aCGH data was used as a systematic correction of artifacts caused by GC content or fragment length^24^. We calculated the QC scores for each sample based on the statistical variance of the probe-to-probe log ratios and removed samples with QC > 0.15. We also removed samples with excessive numbers of autosomal CNVs (subject QC). Then, we excluded CNVs <5 kb, those with >50% overlap with segmental duplications, and those on the Y chromosome (CNV QC). Finally, we filtered out common CNVs (≥1% of the total sample) and identified rare exonic CNVs in *ARHGAP10*.

To evaluate the differences in the frequencies of exonic CNVs in the *ARHGAP10* gene between cases and controls, one-sided Fisher’s exact tests were used. If no variants were observed in a given cell of the 2 × 2 table, the odds ratio was calculated after a 0 cell correction (0.5 was added to all cells) to reduce bias in estimating the OR^25^.

Next, we validated the exonic CNVs in *ARHGAP10* using TaqMan Copy Number Assays (Hs01753683_cn, Hs00968545_cn, Hs04838743_cn and Hs00326850_cn, Hs02356300_cn; Applied Biosystems, Foster City, CA). Experiments were performed in quadruplicate in 384-well plates in conjunction with an ABI 7900HT Real-Time PCR System, and data were collected with an Applied Biosystems SDSv2.4 (Applied Biosystems). Data were analyzed using a manual Ct threshold of 0.2, and an automatic baseline and copy numbers were determined using the CopyCaller software (Applied Biosystems). All genomic locations are given in NCBI build 36/UCSC hg18 coordinates.

### Resequencing analysis of *ARHGAP10* in SCZ patients with exonic CNVs

We next performed Sanger sequencing to identify second hit *ARHGAP10* variants in six patients with SCZ with exonic CNVs (except for Case #3 in which enough DNA was not available). Sequencing was restricted to select exons encoding the key functional domains, namely, PH, RhoGAP, and SH3. Primers were designed using FastPCR software (PrimerDigital Ltd. Helsinki, Finland) against exons 8−12, 13−18, and 22−23 of *ARHGAP10* (NM_024605.3). PCR was performed on genomic DNA using TaKaRa LA Taq polymerase (Takara Bio, Shiga, Japan). Sanger sequencing was performed using BigDye Terminator v.3.1 Cycle Sequencing Kit (Applied Biosystems) and ABI Prism 3130xl Genetic Analyzer (Applied Biosystems). Sequences were then analyzed using the Mutation Surveyor DNA Analysis Software V4.0 (SoftGenetics, State College, PA, USA). All variants identified were confirmed by independent PCR and Sanger sequencing.

To assess the pathogenicity of missense variants identified, two different prediction algorithms were used: PolyPhen2 (http://genetics.bwh.harvard.edu/pph2/) and PMUT^26,27^. To examine the allele frequency of the missense variant (p.S490P) of *ARHGAP10* and its genetic association with SCZ, we performed genotyping of this variant in the second sample set (3649 SCZ cases and 3620 healthy controls) using the Custom TaqMan SNP Genotyping Assay (Applied Biosystems). One-sided Fisher’s exact tests were used for statistical analysis.

### Establishment of iPSCs from subjects and neuronal differentiation

The iPSCs in this study were established from fresh peripheral blood and differentiated into TH-positive neurons as previously reported^28–30^. Cells were cultured in a 5% CO_2_/18%–22% O_2_ atmosphere during all experiments.

### Live imaging

Time-lapse images were obtained using IncuCyte (Essen Bioscience, USA). Sequential neurite and cell body dynamics on the phase contrast images were quantified using the NeuroTrack software module of IncuCyte^28^. The Rho-kinase inhibitor, Y-27632 (Wako, Japan), was added immediately after cell plating.

Other materials and methods are described in Supplementary materials.

## Results

### Detection of exonic CNVs in *ARHGAP10*

**Fig. 1:**
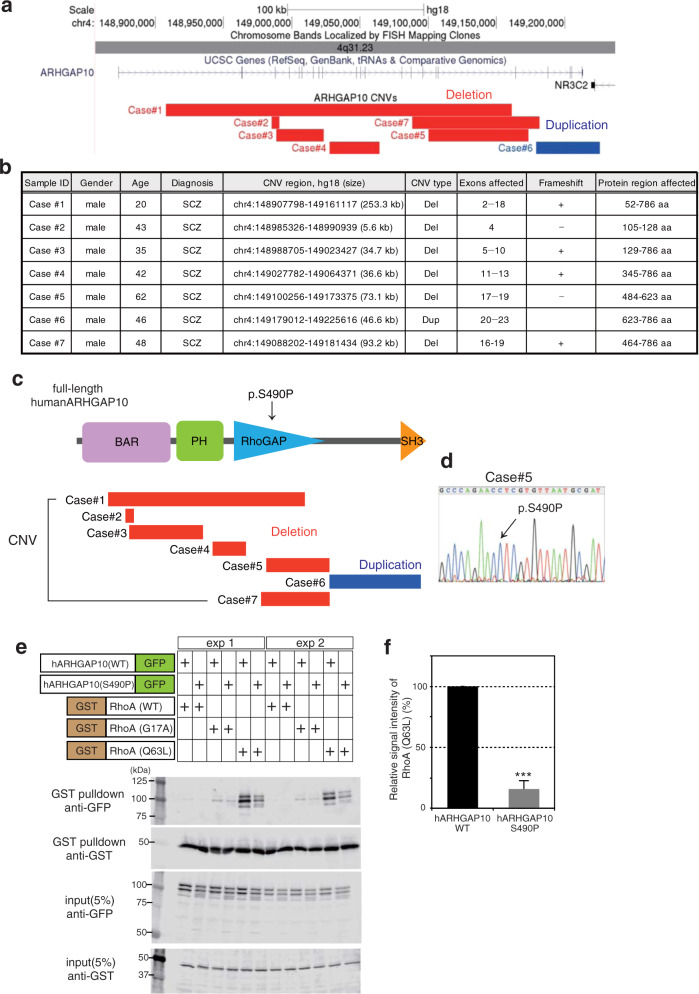
Exonic CNVs in *ARHGAP10* and biological features. **a** The exonic CNVs in *ARHGAP10* identified in this study. Exonic deletions in *ARHGAP10*, indicated by the red boxes, were identified in six patients with SCZ. Exonic duplication, indicated by the blue box, was identified in Case#6. **b** Details of rare exonic CNVs of ARHGAP10 identified in this study. Genomic locations are given in NCBI build 36/UCSC hg18 coordinates. Exons and protein region affected by exonic CNVs are based on NM_024605.3 and NP_078881.3, respectively. **c** The structure of human *ARHGAP10* and the locations of the missense variant (p.S490P) and exonic CNVs identified in patients with SCZ. **d** Sanger sequencing results of the prioritized variant p.S490P. A missense variant (p.S490P) identified in Case#5. This variant was within the exonic deletion of ARHGAP10 on the other allele and located at the RhoGAP domain. **e** GST binding assay using GST-RhoA, GST-RhoA (p.G17A), and GST-RhoA (p.Q63L) as bait proteins and GFP-tagged human ARHGAP10 full-length wild-type (WT) and ARHGAP10 (p.S490P) mutant as pray proteins. Input and bound proteins were detected on immunoblots probed with the anti-GST antibody. Densitometric quantification of immunoblots using Odyssey Clx (LI-COR, USA). **f** Band intensity percentage is presented relative to the corresponding bait (15.8% ± S.E.M. 3.95%, *p* = 0.0002) (three independent experiments).

Of the 3053 patients and 2451 controls analyzed using aCGH, 2940 (96.3%) patients and 2402 (98.0%) controls passed our stringent quality control (QC) filter (Supplementary Fig. 1a). Exonic CNVs in *ARHGAP10* were identified in seven patients (six cases with deletions and one with duplication) (Fig. 1a, b), but not in controls. We then validated all exonic CNVs using the TaqMan Copy Number Assay (Supplementary Fig. 1b). We performed association analysis and found a significant association between exonic CNVs in *ARHGAP10* and SCZ (OR = 12.3, *p* = 0.015).

We then examined the effects of these CNVs on the ARHGAP10 protein. Four of the six exonic deletions were predicted to cause a frameshift, resulting in the truncation of protein regions downstream from the deletion. All exonic CNVs overlapped with functional domain regions of ARHGAP10 (Fig. 1c). Moreover, all exonic deletions were revealed to affect the Bin1/amphiphysin/Rvs167 (BAR) domain, the RhoGAP domain, or both. Breakpoint sequencing further revealed that the exonic duplication in Case #6 was tandem in direct orientation and adjacent to the original locus (Supplementary Fig. 1c).

### Resequencing analysis of *ARHGAP10* in SCZ patients with exonic CNVs

Among the patients with SCZ with exonic CNVs, we identified a missense variant (p.S490P) in exon 17 in one patient (Case #5) (Fig. 1c, d). This variant overlapped with the exonic deletion on the other allele and was located at the RhoGAP domain. PolyPhen-2 and PMUT predictions indicated this variant may be damaging and/or pathological, respectively. The allele frequency of p.S490P was found to be 0.25% and 0.19% in the SCZ sample set and control sample set, respectively, indicating that this variant is rare (<1%) (Supplementary Fig. 1d). No statistically significant association between p.S490P and SCZ was observed (*p* = 0.30).

### Gene expression analysis of *ARHGAP10* in SCZ patients with exonic CNVs

Lymphoblastoid cell lines (LCLs) were established from the peripheral blood of the two patients (Case #4 and #5) with exonic CNVs in *ARHGAP10*. The relative expression levels of *ARHGAP10* mRNA in patients with *ARHGAP10* deletion were significantly decreased compared with those in the schizophrenia group and control group (Supplementary Fig. 1e, f).

### Clinical characteristics of SCZ patients with exonic CNVs in *ARHGAP10*

Table 1 summarizes the clinical data of the seven patients with SCZ (all male) with exonic CNVs in *ARHGAP10*. Three patients had a family history of SCZ or intellectual disability and one patient (Case #5) had poor premorbid functioning (e.g., poor academic performance and social skills) prior to the onset of SCZ. The most frequent psychiatric symptoms were delusions, hallucinations, disorganized speech, and behavior. All patients with clinical data showed a poor response to treatment, with four receiving high doses of antipsychotics (>1000 mg/day chlorpromazine equivalent) at the time of study evaluation.

**Table 1:** Details of clinical characteristics of the patients with rare *ARHGAP10* variants.

	Case #1	Case #2	Case #3	Case #4	Case #5	Case #6	Case #7
Genotype	Exonic deletion in *ARHGAP10*	Exonic deletion in *ARHGAP10*	Exonic deletion in *ARHGAP10*	Exonic deletion in *ARHGAP10*	Exonic deletion and p.S490P in *ARHGAP10*	Exonic duplication in *ARHGAP10*	Exonic deletion in *ARHGAP10*
Diagnosis	SCZ	SCZ	SCZ	SCZ	SCZ	SCZ	SCZ
Age/sex	20/M	43/M	35/M	42/M	62/M	46/M	48/M
Family history	None	ID	None	SCZ	SCZ	None	None
History of development	Normal	NA	Normal	Normal	Poor academic performance and social skills	NA	Normal
Age of onset	18	24	32	37	19	17	20
Main symptoms	Persecutory delusion, social withdrawal	Delusion, hallucination	Disorganized speech and behavior, thought broadcasting, agitation, depressive symptoms	Disorganized speech and behavior, persecutory delusion, auditory hallucination, negative symptoms, cognitive impairment	Delusion, visual hallucination, violent and aggressive behavior, negative symptoms, cognitive impairment	Persecutory delusion, auditory hallucination	Delusion of reference and observation, interpersonal tension
Length of hospitalization	None	19 years	<1 year	4 years	8 years	>20 years	None
Doses of antipsychotics (chlorpromazine equivalent)	NA	2700 mg	480 mg	1300 mg	410 mg	1050 mg	1700 mg
Response to treatment	NA	Poor	Poor	Poor	Poor	Poor	Poor
Comorbidity of physical illnesses	Febrile seizures, benign tumor	None	None	Atrial septal defect	Hypertension, diabetes mellitus, rheumatoid arthritis, psoriasis	Anemia	Hyperlipemia

*ID* intellectual disability, *NA* not available, *SCZ* schizophrenia.

### The *ARHGAP10* p.S490P mutant impairs the interaction with RhoA

Genetic analysis indicated that *ARHGAP10* is a novel candidate gene for SCZ. We focused on one patient (Case #5) who had a “double-hit” of simultaneous mutations, including a CNV and missense variant (p.S490P). Two mutations in the same gene, such as in Case #5, has been proposed to represent a typical genetic model of severe SCZ, as shown in Fig. 1c, d. This patient potentially may express both the ARHGAP10 p.S490P mutant protein and a truncated protein lacking the RhoGAP and SH3 domains.

We next considered the possibility that ARHGAP10 p.S490P lacks Rho-inactivating activity because this variant is located in the RhoGAP domain. To determine the biological significance of ARHGAP10 p.S490P, we analyzed the binding of ARHGAP10 (WT)-GFP and ARHGAP10 (p.S490P)-GFP to GST-RhoA (WT), GST-RhoA (G17A), and GST-RhoA (Q63L) expressed in HEK293 cells (Fig. 1e). In contrast with ARHGAP10 (WT), the protein interaction of the ARHGAP10 p.S490P mutant, with the constitutively active RhoA (RhoA p.Q63L), was found to be significantly decreased (15.8% ± 3.95%, *p* = 0.0002) (Fig. 1f). Thus, the ARHGAP10 p.S490P mutant appeared to attenuate the interaction between ARHGAP10 and RhoA, resulting in the hyperactivation of RhoA.

### Expression profiles of the ARHGAP10 protein in reporter mice

*ARHGAP10* mRNA has been reported to be expressed in the brain^14^. However, the expression profile of the endogenous ARHGAP10 protein (Q6Y5D8-1) has not been fully characterized. Since well-validated ARHGAP10 antibodies are not currently available, we developed *Arhgap10-FLAG* reporter mice harboring a FLAG-tag inserted into the C-terminal side of the *Arhgap10* gene by CRISPR/Cas9 technology^31^. Unexpectedly, the resulting expression level was so weak that it could not be detected without enrichment by immunoprecipitation (Supplementary Fig. 2).

Next, we prepared *Arhgap10-3pV5-mCherry* reporter mice carrying three tandem V5-tags and the mCherry cassette to increase the sensitivity and fluorescent visualization (Supplementary Fig. 3a–c). Quantitative immunoblot assays further revealed the copy number of reporter alleles (Supplementary Fig. 3d), and the ARHGAP10 protein was found to be widely expressed in the brain of the mice at postnatal day 28 (P28) (Supplementary Fig. 3e). The adult brains of two types of knock-in reporter mice were then prepared and concentrated by pull-down using agarose beads conjugated with the V5 tag antibody, and the expression of V5 and the ARHGAP10 protein in the pull-down product was confirmed (Supplementary Fig. 3f, g). In addition, ARHGAP10 was found to be expressed in the brain at E15, P0, and P28 (Supplementary Fig. 3h). Moreover, a short isoform, currently unknown in the mouse database, was detected in the developing brain.

We then prepared dissociated neurons from the reporter mice embryos and detected *ARHGAP10* reporter expression in TH-positive cells (Supplementary Fig. 3i). This reporter expression was more dominant in immature neurons (DIV + 3) than in mature neurons (DIV + 14) (Supplementary Fig. 3j, k).

### Generation of Case #5 model mice and morphological analysis of early neurites

We hypothesized that *ARHGAP10* variants increase SCZ risk. Therefore, we considered that Case #5, exhibiting a severe SCZ phenotype and both an exonic deletion and a rare missense variant, could serve as a novel SCZ model of abnormal brain development. Thus, we generated Case #5 model mice to investigate the molecular basis of SCZ pathogenesis.

**Fig. 2:**
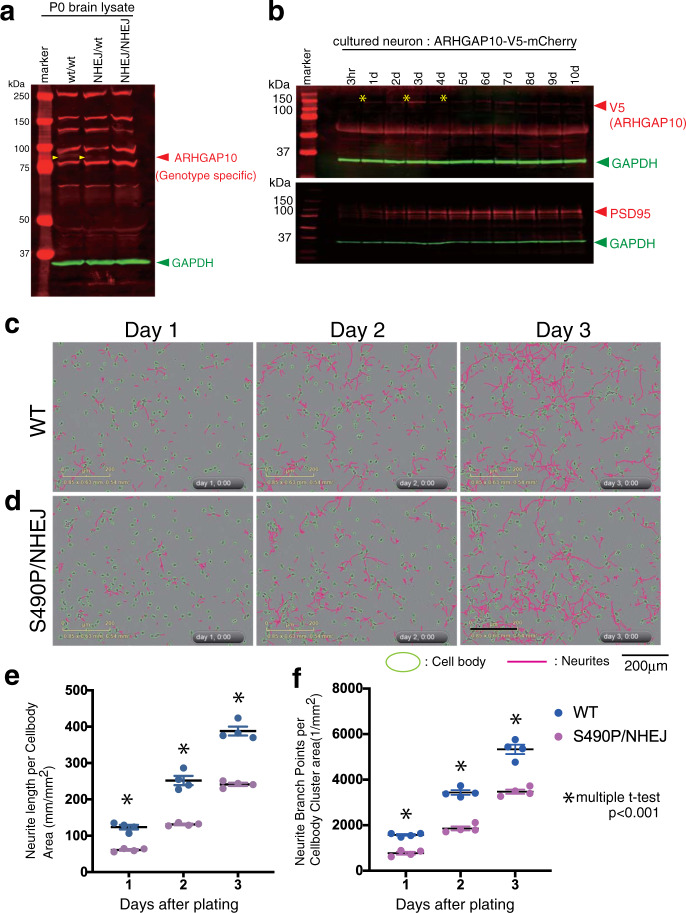
Generating model mice mimicking Case #5 (*Arhgap10* S490P/NHEJ) and time-lapse analysis. **a** Immunoblot analysis to detect endogenous ARHGAP10 in neonatal whole brain lysates. Arrowhead at 90 kDa due to genotype specific signals. Other signals were non-specific. **b** The expression level of endogenous ARHGAP10 associated with the maturation of the primary cultured neurons was analyzed by immunoblotting. Neurons were prepared from the cerebral cortex (E15) of the fetal brain of an *Arhgap10-3pV5-mCherry* knock-in reporter mouse and spread evenly. Lysates were collected immediately after attachment (3 h) and every other day. V5 reflects endogenous ARHGAP10 from the reporter allele. PSD95 is a post-synaptic marker associated with neuronal maturation; ARHGAP10 was found to be strongly expressed in the early stages of culture. **c**, **d** Primary cultured neurons were prepared from the E15 cerebral cortex of wild-type (**c**) and S490P/NHEJ mutant (**d**) embryos and seeded evenly in 4-wells on a PO-coated 12-well culture plate to form neurites during the culture. This process was observed by time-lapse using IncuCyte Zoom (Essen Bioscience, USA), which collected bright field imaging every 30 min. Images were taken continuously at 36 locations in each well, and cell bodies and neurites were automatically detected by the NeuroTrack application in the device. **e**, **f** The total neurite length (**e**) and the number of branches (**f**) at 1, 2, and 3 days after the start of culture were calculated by NeuroTrack and are shown in Supplementary Fig. 4b. Values indicate the mean ± S.E.M. Statistical analysis, with a *t*-test at each time point, showed significant differences between WT and S490P/NHEJ.

We used TALEN (transcription activator-like effector nuclease) genome editing technology to generate mice with variants of *Arhgap10* that mimicked the Case #5 genotype. To this end, we generated Case #5 model mice carrying a missense variant (p.S490P) and a coexisting frameshift mutation caused by non-homologous end-joining (NHEJ) (Supplementary Fig. 4a). Immunoblot analysis did not detect the full-length ARHGAP10 protein (predicted molecular mass, 90 kDa) in lysates prepared from the brains of P0 NHEJ/NHEJ mice, thus confirming a deficiency in the synthesis of full-length ARHGAP10 (Fig. 2a).

Neuronal development is regulated by Rho GTPases activity, which in turn is negatively controlled by RhoGAPs^13^. ARHGAP10 belongs to the RhoGAP family and transduces signals through RhoA that are essential for cytoskeletal organization^14^. However, the contribution of ARHGAP10 to neuronal polarization remains unknown. Using V5 tag knock-in mice, we examined the stage at which the expression of ARHGAP10 became increased (Fig. 2b, upper). The expression was relatively strong for the first three days after the neurons were plated. However, ARHGAP10 expression decreased with neuronal maturation. For these experiments, PSD95 was used as a neuronal maturation marker (Fig. 2b, lower).

To investigate the effects of the *Arhgap10* variants on neurodevelopment, we time-lapsed the morphologies of primary cultures of murine neurons for three days, mimicking stage-3 neurite development. Every day, the total length of the neurites and the total number of bifurcations of the intact neurites cultured in each well on the culture plate were automatically calculated by NeuroTrack software (Fig. 2c, d). As a result, the neurons of the S490P/NHEJ mutant showed a significant reduction in both neurite length and branch number, suggesting that they were more immature (Fig. 2e, f). The results of the statistical analysis are shown in Supplementary Fig. 5b. In the long-term culture (21 days) of neurons from the S490P/NHEJ mutant, the neurons were deemed too fragile to consistently observe synaptic formation.

### Model mouse carrying the genotype of Case #5 exhibited abnormal emotional behavior

**Fig. 3:**
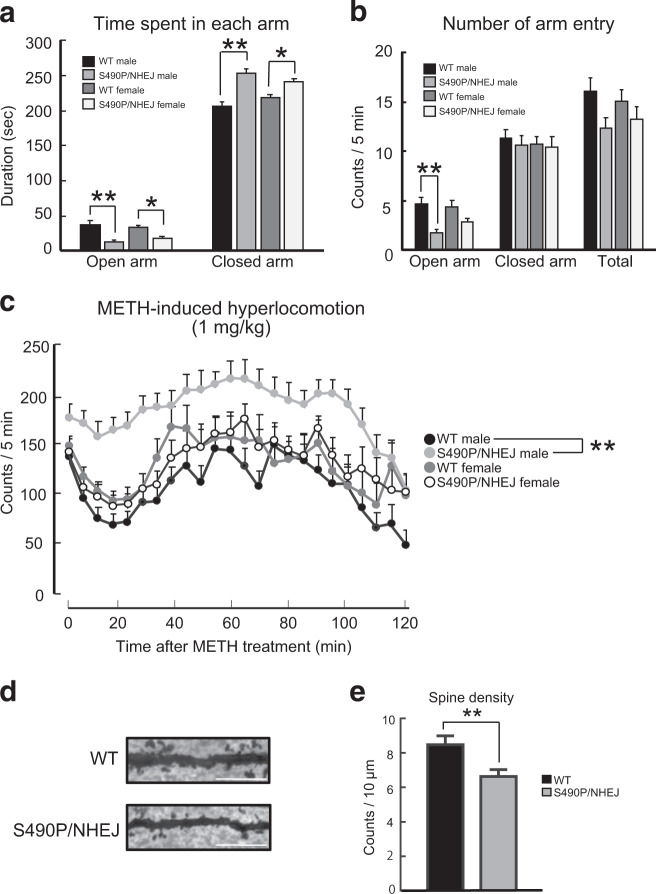
Behavioral abnormalities and changes in dendritic spine density of Case#5 model mice. **a**, **b** Performance in the elevated plus maze test. The time spent in each arm was determined according to genotype and sex. Values indicate the mean ± S.E.M [WT male (*n* = 18), WT female (*n* = 20), *Arhgap10* S490P/NHEJ male (*n* = 21), and *Arhgap10* S490P/NHEJ female (*n* = 21)] (**a**). The number of arm entries was characterized according to genotype and sex. Values indicate the mean ± S.E.M [WT male (*n* = 18), WT female (*n* = 20), *Arhgap10* S490P/NHEJ male (*n* = 21), and *Arhgap10* S490P/NHEJ female (*n* = 21)] (**b**, **c**). Performance of *Arhgap10* S490P/NHEJ mice in the METH (1 mg/kg, i.p.)-induced hyperlocomotion test. Counts/5 min were determined according to genotype and sex. Values indicate the mean ± S.E.M [WT male (*n* = 17), WT female (*n* = 15), *Arhgap10* S490P/NHEJ male (*n* = 19), and *Arhgap10* S490P/NHEJ female (*n* = 15)]. **d** Representative images of the spines of pyramidal neurons in the mPFC (Golgi staining). Scale bar: 10 μm. **e** Quantitative analysis of the dendritic spine density of the cortical pyramidal neurons [*n* = 16 neurons from four mice in each group].

Next, we performed in vivo phenotypic analysis of the Case #5 mouse model with mutations in both *Arhgap10* alleles. To determine the pathophysiological significance of the Case #5 model mice in vivo, *Arhgap10* S490P/NHEJ mice, as well as their WT litter mates, were subjected to behavioral testing. In the elevated plus maze test, two-way ANOVA revealed a significant effect of genotype on the time spent in the open arm [sex, *F* (1, 76) = 0.067, *p* = 0.797; genotype, *F* (1, 76) = 28.5, *p* = 9.5E-07; sex × genotype interaction, *F* (1,76) = 1.72, *p* = 0.194)] and closed arm [(sex, *F* (1, 76) = 0.00219, *p* = 0.963; genotype, *F* (1, 76) = 37.5, *p* = 3.78E-08; sex × genotype interaction, *F* (1,76) = 5.26, *p* = 0.0246; WT male (*n* = 18), WT female (*n* = 20), *Arhgap10* S490P/NHEJ male (*n* = 21) and *Arhgap10* S490P/NHEJ female (*n* = 21)]. Compared with the WT mice, *Arhgap10* S490P/NHEJ mice spent less time in the open arm and remained in the closed arm significantly longer (Fig. 3a). In addition, the number of open-arm entries of *Arhgap10* S490P/NHEJ male mice was lower than that observed for WT male mice [open-arm entry; sex, *F* (1, 76) = 0.765, *p* = 0.384; genotype, *F* (1, 76) = 21.5, *p* = 1.44E-05; sex × genotype interaction, *F* (1,76) = 2.14, *p* = 0.147; closed-arm entry; sex, *F* (1, 76) = 0.229, *p* = 0.634; genotype, *F* (1, 76) = 0.255, *p* = 0.615; sex × genotype interaction, *F* (1,76) = 0.0340, *p* = 0.854; WT male (*n* = 18), WT female (*n* = 20), *Arhgap10* S490P/NHEJ male (*n* = 21) and *Arhgap10* S490P/NHEJ female (*n* = 21)] (Fig. 3b).

In the methamphetamine (METH)-induced hyperlocomotion test, repeated measures three-way ANOVA revealed significant effects of time, genotype, and genotype × sex [time, *F* (23, 1426) = 11.1, *p* = 1.70E−37; genotype × time interaction, *F* (23, 1426) = 0.570, *p* = 0.949; sex × time interaction, *F* (23, 1426) = 0.967, *p* = 0.506; genotype × sex × time interaction, *F* (23, 1426) = 0.667, *p* = 0.881; genotype, *F* (1, 62) = 6.71, *p* = 0.012; sex, *F* (1, 62) = 1.00, *p* = 0.32; genotype × sex interaction, *F* (1, 62) = 6.57, *p* = 0.013; WT male (*n* = 17), WT female (*n* = 15), *Arhgap10* S490P/NHEJ male (*n* = 19) and *Arhgap10* S490P/NHEJ female (*n* = 15)]. The post hoc Tukey test revealed that METH-induced hyperactivity was significantly increased in male *Arhgap10* S490P/NHEJ mice compared with male WT mice (*p* < 0.0001) (Fig. 3c).

No significant differences were revealed from the following tests: open field, light dark box, social interaction, pre-pulse inhibition, Y-maze, novel object recognition, fear conditioning, locomotor activity, and rota-rod (Supplementary Figs. 4c, 5). Collectively, our results suggest that *Arhgap10* S490P/NHEJ mice, particularly male mice, have emotional impairments and are highly sensitive to METH.

### Histochemical analyses of *Arhgap10* S490P/NHEJ mice

To clarify the pathological phenotype of the *Arhgap10* S490P/NHEJ mice, we analyzed the spine density in the medial prefrontal cortex (mPFC) of the male mice, because the mPFC has been reported to be associated with anxiety-related behavior^32,33^. Golgi staining showed that dendritic spine density in the pyramidal neurons of layers II/III was significantly decreased in *Arhgap10* S490P/NHEJ male mice compared with WT male mice [*t*(30) = 2.835, *p* = 0.00812 (*n* = 16 neurons from four mice in each group); Fig. 3d, e], suggesting that *Arhgap10* may be involved in the regulation of spine density in vivo. Images of Nissl, NeuN, GFAP, and Iba1 staining in the mPFC, striatum, and hippocampus were similar between *Arhgap10* S490P/NHEJ male mice and WT male mice (Supplementary Fig. 6).

### In vitro morphological analysis of neurons differentiated from Case #5 iPSCs

As previously demonstrated, the ARHGAP10 protein was found to be expressed in cultured neurons prepared from fetal mouse brain and was shown to affect neurogenesis. While the histopathological phenotype of Case #5 model mice in vivo was minor (Supplementary Fig. 6), general behavioral tests revealed some phenotypes that were considered similar to SCZ (Fig. 3). Therefore, we next established Case #5 iPSCs to further determine whether *ARHGAP10* variants are associated with neurodevelopmental disorders in humans, and compared the in vitro morphological phenotypes related to neurite elongation and maturation between Case #5 and healthy controls.

**Fig. 4:**
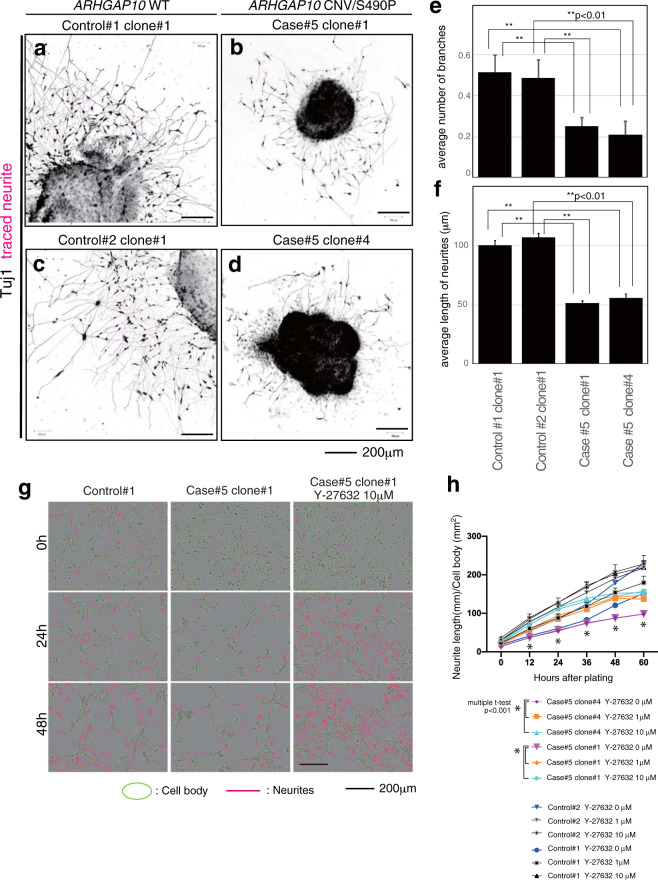
Morphological analysis of Case #5 (*ARHGAP10* CNV/SNV) neurons. **a**–**f** In vitro morphological analysis of human neurons derived from iPSCs; Control #1 (**a**), Control #2 (**c**), Case #5, clone #1 (**b**), Case #5, clone #4 (**d**). **e** Average number of branches on the primary neurites (Control #1, 0.514 ± 0.086, *n* = 72; Control #2, 0.487 ± 0.087, *n* = 78; Case #5, clone #1, 0.252 ± 0.040, *n* = 147; Case #5, clone #4, 0.209 ± 0.054, *n* = 67; *t*-test *P* values: Control #1 vs Case #5, clone #1, *p* = 0.0068; Control #1 vs Case #5, clone #4, *p* = 0.0034; Control #2 vs Case #5, clone #1, *p* = 0.0153; Control #2 vs Case #5, clone #4, *p* = 0.0076). **f** Average length of primary neurites (Control #1, 116.3 ± 4.29 μm, *n* = 382; Control #2, 126.2 ± 3.81 μm, *n* = 562; Case #5, clone #1, 59.0 ± 1.88 μm, *n* = 575; Case #5, clone #4, 65.7 ± 3.82 μm, *n* = 252; *t*-test *P* values: Control #1 vs Case #5, clone #1, *p* = 0.0025; Control #1 vs Case #5, clone #4, *p* = 0.0023; Control #2 vs Case #5, clone #1, *p* = 0.0002; Control #2 vs Case #5, clone #4, *p* < 0.0001). **g** Two iPS cell clones derived from healthy controls and two iPS cells derived from Case #5 were differentiated into neurons, dispersed, and cultured on a plate. Time-lapse observation was then performed every 12 h and imaged for 60 h after seeding at three concentrations (0, 1, and 10 μM) of the Rho-kinase inhibitor, Y-27632. **h** A graph obtained by analyzing the image data of (**g**) the automatic tracking with NeuroTracker in IncuCyte Zoom. Culture time was plotted on the horizontal axis, while neurite outgrowth was plotted on the vertical axis. Statistical analyses are shown in Supplementary Fig. 7b. Values indicate the mean ± S.E.M.

We established two iPSC clones from the Case #5 subject (Case #5, clone #1 and Case #5, clone #4) and one iPSC clone from each healthy subject (Control #1, clone #1 and Control #2, clone#1), confirming their quality for pluripotency (Supplementary Fig. 7a). We then differentiated the cells into dopaminergic-like neurons via neurosphere formation^28^. Nearly all the neurons obtained using this differentiation method were identified as TH-positive neurons. Although ARHGAP10 protein expression in the human TH-positive neurons could not be definitively confirmed due to the insufficient sensitivity of the ARHGAP10 antibody, they were considered to be ARHGAP10-positive since *Arhgap10* was expressed in mouse TH-positive neurons, as shown in Supplementary Fig. 3i. When we further analyzed Case #5 neurons, we found that the average number of branching points during the early stage of neurite elongation with Case #5 in vitro was significantly lower than that of the two controls (Control #1, 0.514 ± 0.086, *n* = 72; Control #2, 0.487 ± 0.087, *n* = 78; Case #5, clone #1, 0.252 ± 0.040, *n* = 147; Case #5, clone #4, 0.209 ± 0.054, *n* = 67; *t*-test *P* values: Control #1 vs Case #5, clone #1, *p* = 0.0068; Control #1 vs Case #5, clone #4, *p* = 0.0034; Control #2 vs Case #5, clone #1, *p* = 0.0153; Control #2 vs Case #5, clone #4, *p* = 0.0076) (Fig. 4a–e). In addition, the average *i*n vitro length of the neurites of Case #5 was significantly shorter (Control #1, 116.3 ± 4.29 μm, *n* = 382; Control #2, 126.2 ± 3.81 μm, *n* = 562; Case #5, clone #1, 59.0 ± 1.88 μm, *n* = 575; Case #5, clone #4, 65.7 ± 3.82 μm, *n* = 252; *t*-test *P* values: Control #1 vs Case #5, clone #1, *p* = 0.0025; Control #1 vs Case #5, clone #4, *p* = 0.0023; Control #2 vs Case #5, clone #1, *p* = 0.0002; Control #2 vs Case #5, clone #4, *p* < 0.0001) (Fig. 4a–d, f). Thus, our in vitro morphological analysis revealed that the human TH-positive neurites with the *ARHGAP10* CNV/SNV genotype were impaired both in length and branching.

Next, we examined whether the shortening of the process extension and the decrease in branching of the neurons differentiated from Case #5 iPSCs were due to activated Rho-kinase caused by decreased RhoGAP activity associated with the *ARHGAP10* variants. To this end, the Rho-kinase inhibitor, Y-27632, was added immediately after plating the neurosphere of Case #5-derived iPSCs, and neurite elongation was imaged every 12 h for 60 h, with neurite length automatically quantified (Fig. 4g)^34^. For the two clones of Case #5 (clones #1 and #4), the neurite lengths without the presence of Y-27632 and with 1 or 10 μM Y-27632 were compared at each point. Our results indicated that neurite elongation was significantly increased by the addition of 1 or 10 μM Y-27632 at all time-points and was recovered to similar levels as observed in the healthy control (Fig. 4h; Supplementary Fig. 7b).

## Discussion

In the present study, we found exonic CNVs of *ARHGAP10* in seven patients with SCZ, which provided evidence for a genetic association with SCZ (OR = 12.3, *p* = 0.015). *ARHGAP10* encodes a Rho GTPase-activating protein that catalyzes the conversion of active GTP-bound Rho GTPases to their inactive GDP-bound form, thus suppressing various Rho GTPase-mediated cellular processes. Rho GTPases regulate numerous cell functions, including cell cytoskeleton organization, migration, gene transcription, adhesion, cellular proliferation, and survival^21,35^. Although *ARHGAP10* itself has not been associated with SCZ in the literature, small GTPase signaling has been implicated in the pathogenesis of SCZ^2,3,5^. All CNVs identified in the SCZ patients overlapped with the known functional domains of *ARHGAP10* (BAR, PH, RhoGAP, and SH3 domains), suggesting a damaging effect on protein function. Expression analysis using LCLs from two patients (Case #4 and #5) also suggested that exonic CNVs result in a lower level of *ARHGAP10* mRNA.

Interestingly, we identified a “double-hit” patient (Case#5) who carried an exonic deletion and a deleterious missense variant (p.S490P) in each *ARHGAP10* allele. The co-occurrence of such variants in the same gene has recently been suggested as an important genetic mechanism of SCZ^23^. In other words, the discovery of such a double-hit event in *ARHGAP10* provides additional support for the involvement of this gene in SCZ. Therefore, we considered that Case #5 would have more severe pathophysiology related to *ARHGAP10* defects. In fact, Case #5 had premorbid cognitive dysfunction (e.g., poor academic performance and social skills) as well as severe psychiatric symptoms that required long-term hospitalization. Thus, we focused on Case #5 for the in depth analysis in this study. We demonstrated that the residue at S490 in the RhoGAP domain of the ARHGAP10 protein contributed to the binding of RhoA (Fig. 1f, g). RhoA is a negative regulator of numerous functions, including those that control axonal decisions^1,36^.

The p.S490P variant likely contributes to the loss of neural polarity as a result of decreased binding of ARHGAP10 to RhoA. We hypothesized that the coexistence of the ARHGAP10 deletion and the missense variant is associated with the molecular pathogenesis of mental disorders. As evidence of this, a genetically modified mouse model mimicking the Case #5 genotype showed impaired neurite elongation (Fig. 2) and behavioral phenotypic abnormalities (Fig. 3). The phenotypes of these mice were considered to be linked to the clinical findings of Case #5 and the neurite impairment observed in Case #5 iPSCs (Fig. 4).

The *Arhgap10* S490P/NHEJ mice we created in this study may prove to be a valid SCZ model. For example, anxiety-like behavior in the elevated plus maze test was increased in the *Arhgap10* S490P/NHEJ mice compared with their WT litter mates. Male, but not female, *Arhgap10* S490P/NHEJ mice were more sensitive to METH, which is consistent with enhanced dopamine release following amphetamine challenge in SCZ patients during acute illness^37^. In addition to these behavioral changes, spine density in the pyramidal neurons of the mPFC was shown to be significantly reduced in male *Arhgap10* S490P/NHEJ mice. Previous studies have demonstrated a link between anxiety-like behaviors and morphological changes in the mPFC^38–40^. These findings suggest that the emotional abnormalities observed in the *Arhgap10* S490P/NHEJ male mice may be associated with changes in spine density in the mPFC. These sex differences may be comparable to the general clinical finding that male SCZ patients have an earlier onset, worse premorbid functioning, and poorer prognoses than female patients, despite the fact that similar incidences of SCZ exist between men and women^41,42^. These sex differences have been hypothesized to be attributed, at least partly, to sex hormones, with female sex hormones such as oestradiol acting as antipsychotics when administered to SCZ patients^43^.

Although we demonstrated ectopic dendritic formation in the *Arhgap10* S490P/NHEJ mice, changes in cellular architecture and populations of the mPFC, hippocampus, and striatum were considered minimal (Supplementary Fig. 6). We hypothesize that the p.S490P variant and frameshift mutations (NHEJ) inactivate the ARHGAP10 protein. In addition, it is known that RhoA signaling mediates synapse formation^44^ and our results indicated that neuronal synapse formation was attenuated in Case #5 model mice (Fig. 3d, e). This may be related to the lack of ARHGAP10 activity early in neurite formation (Fig. 2b). Therefore, the role of ARHGAP10 in both synapse formation during early development and synaptic function in adulthood needs to be fully addressed in the future. In addition, RhoA signaling may contribute to neurotransmission, and activation of the RhoA signal transduction pathway decreases the phosphorylation of the GABA_A_ receptor^45^ and mediates amphetamine-induced dopamine transporter (DAT) internalization^46^. However, the question of whether *ARHGAP10* mutations contribute to GABAergic and dopaminergic dysfunction remains to be determined.

In this study, we generated novel *ARHGAP10* reporter mice to clarify the expression pattern of the ARHGAP10 protein in the brain and cultured neurons (Supplementary Fig. 3). To detect the endogenous ARHGAP10 protein, three V5 tags were inserted and tandemly repeated in-frame, with the expression confirmed in the brain and primary cultured neurons by immunoblot analysis. However, immunostaining with brain slices failed to detect specific signals against anti-V5; thus, the expression level of the ARHGAP10 protein should be extremely low. Furthermore, in reporter mice, our results suggested that short isoforms were specifically present in the fetal brain (Supplementary Fig. 3h). Although these short isoforms have not been reported in mice, a similar isoform of ARHGAP10 has been registered in the Ensembl genome database (transcription: ARHGAP10-204). Our reporter mice should be useful for estimating where the ARHGAP10 protein is expressed in the brain and where ARHGAP10 carries out important functions if relevant immunostaining tools and conditions can be improved upon.

The expression of the ARHGAP10 protein in cultured neurons was shown to be strong early in neurite formation (Fig. 2b). We then observed the primary cultured neurons from *Arhgap10* S490P/NHEJ embryos at the early stage of neurodevelopment. As a result, the neurites of the primary cultured neurons isolated from the *Arhgap10* S490P/NHEJ mice had shorter neurites and fewer branches than the WT mice (Fig. 2c–f). These observations support the conclusion that, in neurons from *ARHGAP10* S490P/NHEJ mice, RhoA is excessively activated, which serves to inhibit neurite outgrowth during early neurodevelopment^47^. However, other members of the RhoGAP family should co-exist in neurons; thus, there is a possibility that local RhoA activity may be altered by the subcellular localization of ARHGAP10. Future research to better understand the expression of the ARHGAP10 protein, including its intracellular localization, is warranted in order to elucidate the mechanism of pathogenesis caused by *ARHGAP10* mutations.

In this study, we had the unique opportunity to establish iPSCs from the Case #5 subject. Indeed, human iPSCs have been recently established as an induction system to differentiate into various subtypes of neurons and appear to be promising bioresources for clinical research. We analyzed the morphological phenotype of TH-positive neurons derived from the Case #5 iPSCs and demonstrated that they showed similar impairment as the cultured neurons from the Case #5 mouse model. It is assumed that there would be common molecular mechanisms with regard to Rho signaling between human and mouse because *ARHGAP10* is highly conserved in both species (human, NP_078881.3; mouse, NP_084389.2). Further determination of the distribution and function of the ARHGAP10 protein in neurons using Case #5 model mice/iPSCs may provide greater understanding of the relationships that exist between genetic variation and function and the etiology of the pathological and behavioral phenotypes observed in the patients and model mice.

There were some limitations in this study. First, the lack of sensitive and highly specific antibodies recognizing the endogenous ARHGAP10 protein and the very low gene expression levels made it difficult to fully reveal the subcellular localization of ARHGAP10. Second, the expression of the ARHGAP10 wild-type and mutants encoded by the *ARHGAP10* CNV deletion allele or the *ARHGAP10* p.S490P mutant allele into the mouse brain/neurons was too difficult to detect itself because of the low expression and high cytotoxicity of the plasmids. Third, the knockout allele in the mouse is a protein deletion due to a 10-bp deletion frameshift (Supplementary Fig. 4a). Therefore, the mouse model does not completely mimic the deletion of Case #5 and may reflect effects other than the deletion or mutation of the ARHGAP10 protein. Fourth, we were unable to generate isogenic iPSCs mimicking Case #5 because they would need to include two different mutations, namely, the p.S490P knock-in and the deletion. Finally, it remains necessary to establish a long-term culture system of neurons derived from the model mice, as well as iPSCs that are vulnerable to neurite formation, to clarify the synaptic pathology of *ARHGAP10* mutations.

In summary, we identified *ARHGAP10* as a novel risk gene for SCZ. In addition, we successfully generated *Arhgap10* S490P/NHEJ model mice from Case #5, which exhibited severe SCZ phenotypes. Furthermore, we established iPSCs from Case #5, revealing a similar impairment of neurite development to primary cultured neurons from Case #5 model mice. For future research, in order to clarify the function of ARHGAP10 in terms of neuronal polarity, it may be effective to use Case #5 model and reporter mice to analyze the sequential activities of cellular RhoA and/or Cdc42 as ARHGAP10 substrates. If this investigation could be reproduced in Case #5 iPSC-derived neurons, it may lead to the discovery of a compound clinically better than Y-27632 that provides a rescue effect for neuronal polarity in Case #5 model mice and which could potentially be applied as part of a novel clinical treatment for SCZ.

## Supplementary information

Supplementary materials

Supplementary figure 1

Supplementary figure 2

Supplementary figure 3

Supplementary figure 4

Supplementary figure 5

Supplementary figure 6

Supplementary figure 7
