# Supplementary material for: ARHGAP10, which encodes Rho GTPase-activating protein 10, is a novel gene for schizophrenia risk
Source: Transl Psychiatry. 2020 Jul 22;10:247. doi: 10.1038/s41398-020-00917-z (PMC7376022; doi:10.1038/s41398-020-00917-z)
Supplement: Supplementary file 1 — Supplementary materials [file 41398_2020_917_MOESM1_ESM.docx]

**Supplementary materials**

***ARHGAP10*, which encodes Rho GTPase-activating protein 10, is a novel gene for schizophrenia risk**

**Authors:**

Mariko Sekiguchi^1,3,†^ , Akira Sobue^2,†^, Itaru Kushima^1, 15, †^, Wang Chenyao^1, †^, Yuko Arioka^1,4,†^, Hidekazu Kato^1^, Akiko Kodama^1,3^, Hisako Kubo^1^, Norimichi Ito^2^, Masahito Sawahata^2^, Kazuhiro Hada^2^, Ryosuke Ikeda^1,2,3^, Mio Shinno^1,2,3^, Chikara Mizukoshi^1^, Keita Tsujimura^1^, Akira Yoshimi^1^, Kanako Ishizuka^1^, Yuto Takasaki^1^, Hiroki Kimura^1^, Jingrui Xing^1^, Yanjie Yu^1^, Maeri Yamamoto^1^, Takashi Okada^1^, Emiko Shishido^1^, Toshiya Inada^1^, Masahiro Nakatochi^14^, Tetsuya Takano^3^, Keisuke Kuroda^3^, Mutsuki Amano^3^, Branko Aleksic^1^, Takashi Yamomoto^5^, Tetsushi Sakuma^5^, Tomomi Aida^6^, Kohichi Tanaka^6^, Ryota Hashimoto^7,8,9^, Makoto Arai^10^, Masashi Ikeda^16^, Nakao Iwata^16^, Teppei Shimamura^11^, Taku Nagai^2^, Toshitaka Nabeshima^12^, Kozo Kaibuchi^3^, Kiyofumi Yamada^2,*^, Daisuke Mori^1,3,13,*^, Norio Ozaki^1^

**Affiliations:**

^1^ Department of Psychiatry, Nagoya University Graduate School of Medicine, Nagoya, Aichi, Japan

^2^ Department of Neuropsychopharmacology and Hospital Pharmacy, Nagoya University, Graduate School of Medicine, Nagoya, Aichi, Japan

^3^ Department of Pharmacology, Nagoya University Graduate School of Medicine, Nagoya, Aichi, Japan

^4^ Center for Advanced Medicine and Clinical Research, Nagoya University Hospital, Nagoya, Aichi, Japan

^5^ Division of Integrated Sciences for Life, Graduate School of Integrated Sciences for Life, Hiroshima University, Hiroshima, Japan

^6^ Laboratory of Molecular Neuroscience, Medical Research Institute, Tokyo Medical and Dental University, Tokyo, Japan

^7^ Department of Pathology of Mental Diseases, National Institute of Mental Health, National Center of Neurology and Psychiatry, Kodaira, Tokyo, Japan

^8^ Molecular Research Center for Children’s Mental Development, United Graduate School of Child Development, Osaka University, Suita, Osaka, Japan

^9^ Department of Psychiatry, Osaka University Graduate School of Medicine, Suita, Osaka, Japan

^10^ Department of Psychiatry and Behavioral Sciences, Tokyo Metropolitan Institute of Medical Science, Tokyo, Japan

^11^ Division of Systems Biology, Nagoya University Graduate School of Medicine, Nagoya, Aichi, Japan

^12^ Advanced Diagnostic System Research Laboratory Fujita Health University, Graduate School of Health Sciences & Aino University, Toyoake, Aichi, Japan

^13^ Brain and Mind Research Center, Nagoya University, Nagoya, Aichi, Japan

^14^Division of Data Science, Department of Nursing, Nagoya University Graduate School of Medicine, Nagoya, Aichi, Japan

^15^ Medical Genomics Center, Nagoya University Hospital, Nagoya, Aichi, Japan

^16^Department of Psychiatry, Fujita Health University School of Medicine, Toyoake, Japan

**Additional Material and Methods:**

**Evaluation of clinical characteristics**

From medical records, we obtained information on clinical characteristics of seven patients carrying rare exonic CNVs of *ARHGAP10*. Characteristics included family history, history of development, age of onset, psychiatric symptoms, length of hospitalization, doses of antipsychotics, response to treatment, and comorbidity of physical illnesses.

**Plasmids constructs**

The cDNA encoding full-length ARHGAP10 (UniProtKB: A1A4S6) was cloned from a human hippocampus cDNA library (Clontech) and inserted into the pEGFPN3 vector (Clontech). ARHGAP10-S490P cDNA was generated using site-directed mutagenesis. pEF-BOS-GST-RhoA and its derivatives G17A and Q63L were gifts from Dr. Kaibuchi.^1^

***In vitro* binding assays**

*In vitro* binding assays were performed as previously described.^2^ Each plasmid was used to transfect HEK293FT cells in the presence of Lipofectamine 2000 (Invitrogen, USA), cultured for 40 h, and lysed in ice-cold buffer containing 20 mM HEPES-NaOH pH 7.5, 150 mM NaCl, 1% Triton-X100, 5 mM MgCl_2_, 1 mM DTT, and a protease inhibitor cocktail (Roche, Germany). Lysates prepared from cells expressing GST-RhoA were bound to glutathione-sepharose 4B beads, which were then incubated for 1 h at 4 °C, with lysates prepared from cells expressing ARHGAP10-GFP. The beads were washed three times with lysis buffer, suspended in SDS-PAGE sample buffer, and the eluted proteins were subjected to immunoblot analysis using anti-GST or anti-GFP antibodies.

**Gene expression analysis**

Lymphoblastoid cell lines (LCLs) derived from subjects with and without *ARHGAP10* deletion were established by widely used Epstein-Barr virus (EBV) transformation with minor modification. ^3^ Total RNA was extracted from LCLs using RNAqueous™ Total RNA Isolation Kit (Thermo Fisher Scientific, Waltham, MA, USA) and treated with DNase to remove contaminated genomic DNA using TURBO DNA-*free*™ Kit (Thermo Fisher Scientific), then reverse transcribed to cDNA with High-Capacity RNA-to-cDNA™ Kit (Thermo Fisher Scientific). Two housekeeping genes, beta-2-microglobulin (*B2M*) and glucuronidase-beta (*GUSB*), were selected as internal control genes to normalize the polymerase chain reaction (PCR). Quantitative real-time PCR (qPCR) was performed on an ABI prism 7900HT Real-Time PCR System (Thermo Fisher Scientific) using predesigned TaqMan Gene Expression Assay probes (Hs01016850_m1 for *ARHGAP10*, Hs99999907_m1 for *B2M* and Hs99999908_ml for *GUSB*; Thermo Fisher Scientific). Measurement of the cycle threshold was implemented in duplicate. The data, including amplifying efficiency and relative expression on quantification, were analyzed using the comparative cycle threshold (Ct) method. ^4^ Expression levels in subjects with *ARHGAP10* deletion (Case#4 and#5) were compared with those in schizophrenia group or control group without *ARHGAP10* deletion, and statistical significance was determined with a two-sided Z test. The significance level was set at 0.05.

**Generating *Arhgap10*-reporter mouse by CRISPR/Cas9 system**

A pair of oligo DNAs (Invitrogen, USA) corresponding to *Arhgap10* sgRNA was hybridized and ligated using T4 DNA ligase (TOYOBO, Japan) into linearized pSpCas9 (BB) -2A-GFP (PX458) plasmid (Addgene, # 48138; Feng Zhang, MIT) digested with BbsI (NEB, USA) as previously described.^5, 6^ DNA primers are listed in Supplementary Figure 2e.

T7E1 assay using mouse Neuro2A cells was performed as described previously.^7^ Briefly, *Arhgap10*-pX458 or empty pX458 plasmids were respectively transfected into Neuro2A cells in a 12-well plate using Lipofectamine 3000 (Life Technologies). After 48 hours post-transfection, genomic DNAs were parepared by proteinase K (P2308, Sigma-Aldrich, USA) digestion and ethanol precipitation. Then, *Arhgap10* loci were PCR amplified from the purified genomic DNA with primers (Supplementary Figure 2e). PCR products were slowly annealed and digested with T7E1 enzyme at 37 °C for 30 minutes and analyzed by electrophoresis in 2% agarose gel.

The crRNA and tracrRNA in this study were chemically synthesized as followed. *Arhgap10*-CRISPR RNA (crRNA) (5′- AACCAGAUUUAGAACGAAAAAGGguuuuagagcuaugcuguuuug- 3′) and trans activating RNA crRNA (tracrRNA) (5′- AAACAGCAUAGCAAGUUAAAAUAAGGCUAGUCCGUUAUCAACUUGAAAAAGUGGCACCGAGUCGGUGCU-3′) were chemically synthesized and purified by polyacrylamide gel electrophoresis (Fasmac, Atsugi, Kanagawa, Japan).

Microinjection was performed as follows. The mixtures for injection were prepared according to previous method.^8^ For *Arhgap10* reporter mouse production by protein injection, Cas9 proteins, *Arhgap10*-crRNA and tracrRNA, and donor double-strand DNA (dsDNA) were mixed in 0.1 TE buffer (Nakarai, Japan) to a working concentration of 100 ng/μl, 0.61 pmol/μl, 0.61 pmol/μl, and 10 ng/μl, respectively. Cas9 proteins were purchased from NEB (M0386S, USA). Donor dsDNA, coding three tandem V5 tag and mCherry gene casette, was chemically synthesized by Fasmac Inc. (Japan). The mixture was incubated at 37°C for at least 15 minutes, and then injected into pronuclei of one-cell-stage zygotes obtained from C57BL/6J strain (Charles River, USA).

For the purpose of PCR screening of Arhgap10 reporter littermates born from the zygotes injected with CRISPR mix. Their genomic DNAs were prepared from tails by proteinase K digestion and a subsequent standard phenol extraction method. *Arhgap10* reporter were screened by PCR with KOD FX Neo (TOYOBO) and analyzed by electrophoresis in 2% agarose gel. PCR primers are listed in Supplementary Figure 2e. PCR products were further cloned with Zero Blunt TOPO PCR Cloning Kits (Life Technologies) and analyzed by sequencing as describe previously.^8^

**Generation of model mouse using the Platinum TALEN method**

The Platinum TALEN plasmids were constructed using the Platinum Gate TALEN Kit (Addgene) as described previously^7^ with some modifications. Briefly, synthesized TALE repeats were cloned into pBluescript SK and assembled using the Golden Gate cloning method.^9, 10^ The modified destination vector harboring the NT-βN mutation^9^ was used. The target sequences of right and left Platinum TALENs were as follows: right, 5'-ACCAAGAAGTGGATGGC-3' and left, 5'-AAGAGAGCGGCAGCCCG-3'. Platinum TALEN mRNAs were synthesized from plasmids linearized using SmaI with an mMessage mMachine T7 Ultra Kit (Life Technologies) and were purified using a MEGAclear kit (Thermo Scientific), following the manufacturers’ instructions and as previously described.

Each Platinum TALEN mRNAs or ssODN donor (TCTTTTCCTTCTTAAAAAAAAAGAGAGCGGCAGCCCGGAACCTCGAGTTAATGCCATCCACTTCTTGGTACACAAACTGCCAG, FASMAC) were diluted and mixed in 0.1TE buffer [10 mM Tris-HCl, 0.1 mM EDTA (pH 8.0)] to a working concentration of 4 ng/μl and 15 ng/μl, respectively. Embryos were obtained by mating of C57BL/6J males and females (CLEA Japan). Microinjection was performed as previously described^6^. After incubation at 37°C for 24 hours, two-cell embryos were transferred into pseudopregnant ICR female mice (CLEA Japan).

Genomic DNA was extracted from the tail of each pup using a DNeasy Blood & Tissue Kit (Qiagen). Genotyping PCR was performed using KOD FX neo DNA polymerase (TOYOBO, Japan) under the conditions as follows: 96 °C for 2 min, followed by 35 cycles at 96 ºC for 15 s, 60 °C for 30 s, and 68 °C for 30 s. The PCR primers were as follows: line p. 490 S<P knock-in (KI) allele, 5'-catgcatgaagctgtgtgagaggtac-3' and 5'-caccgagctgctggcagaggtgtaagcc-3'; WT allele, 5'-gcgagtgccatgtgagctacaag-3' and 5'-gaagtggatggcattaactcggg-3'; line NHEJ allele, 5'-gcgagtgccatgtgagctacaag-3' and 5'-aagaagtggatggcacttccggg-3'. The PCR fragment of line KI was digested with XhoI enzyme (TOYOBO).

**Immunoblots**

For immunoblotting, whole brain extracts were lysed with SDS-lysis buffer (4 % SDS, 20 % glycerol and 50 mM Tris-HCl (pH 6.8) and sonicated on ice. The lysates were diluted to 5 μg/μl. For loading, an equal volume of a solution of 0.01 % bromophenol blue and 1M 2-mercaptoethanol was added to the extracts which were boiled for 3 min at 96 °C. A unit of 50 μg of denatured proteins was loaded for each condition and separated on SDS gradient 5–20 % polyacrylamide gels (e-PAGEL, ATTO, Japan) and transferred on to nitrocellulose membrane (Protran, Whatman). The membrane was probed with the ARHGAP10 antibody (rabbit polyclonal, Sigma-Aldrich, USA) and V5-tag antibody (mouse monoclonal, MCA1360GA, BioRad), following appropriate donkey secondary antibodies, IRDye 680RD and 800CW (LI-COR Biosciences). An infrared imager was used for detection (Odyssey, LI-COR Biosciences). Digital data were exported to TIFF format.

**Animals**

*Arhgap10* mutants (ARHGAP10 KI/NHEJ) as a model of Case #5 in inbred strains of mice with C57BL/6J background were used. S490P/NHEJ mice were generated by intercrossing S490P/wt and NHEJ/wt mice. WT littermates were used as controls. *Arhgap10* KI/NHEJ and WT were 10–15 weeks old when used in all experiments. All of the animal protocols were approved by the Animal Care and Use Committee of Nagoya University Graduate School of Medicine; in addition, the Principles for the Care and Use of Laboratory Animals, which were approved by the Japanese Pharmacological Society, and the National Institutes of Health Guide for the Care and Use of Laboratory Animals were followed.

**Nissl staining**

Mice were deeply anesthetized with Tribromoethanol (Avertin) and perfused intracardially with 0.1 M phosphate-buffer (PB) and 4 % paraformaldehyde in PB. The brains were removed, post-ﬁxed with the same ﬁxative and cryoprotected with 30% sucrose containing PB. Twenty micrometers thick coronal brain sections were cut on a cryostat and mounted on slides. Nissl staining was done according to standard procedures.^11^ Nissl stained sections were analyzed using a light microscope (FSX100; OLYMPUS, Tokyo, Japan).

**Immunostaining**

Immunostaining was conducted as previously described with minor modifications.^12^ Mice were deeply anesthetized with tribromoethanol (Avertin) and perfused intracardially with 0.1 M phosphate-buffer (PB) and 4% paraformaldehyde in 0.1 M PB. The brains were removed, post-ﬁxed with the same ﬁxative and cryoprotected with 30% sucrose containing PB. Twenty micrometers thick coronal brain sections were cut on a cryostat. These slices were fixed with 4% paraformaldehyde in 0.1 M PB for 5 min and then permeabilized with 0.1% Triton X-100/PBS for 10 min. After incubation in blocking solution (5% goat serum/PBS) for 30 min, mouse anti-GFAP (1:300; Sigma-Aldrich, St. Louis, MO, USA), rabbit anti-Iba1 (1:300; Wako, Osaka, Japan), mouse anti-NeuN (1:300; CHEMICON INTERNATIONAL), antibodies diluted in blocking solution were applied to the slices, which were then incubated overnight at 4 ºC. After washing in PBS, goat anti-mouse Alexa Fluor (AF) 488 and anti-rabbit AF488 antibodies (1:1,000; Invitrogen, Eugene, OR) were added to the sections for 1 h at room temperature. After washing in PBS, secondary antibodies were applied. These slides were mounted on slides and observed under a Nikon confocal laser microscope (A1Rsi; Nikon, Tokyo, Japan).

**Golgi staining and morphological analysis**

Golgi staining was carried out by using the FD Rapid Golgi Stain Kit according to the manufacturer’s protocol (FD NeuroTechnologies, Ellicott City, MD) and a previous study. ^13^ The cryosections were sliced at 80 μm using a cryostat. BZ9000 bright-field microscopic (KEYENCE) images (at 100 × magnification) of layers II/III frontal cortex pyramidal neurons were obtained. Only fully impregnated neurons displaying dendritic trees without obvious truncations and isolated from neighboring impregnated neurons were retained for analyses. We quantified the spine density, which was limited to dendrites 30 to 120 μm from the soma. Spine density was expressed as the number of spines per 10 μm of dendrite length (Sobue et al., 2018). All dendrites and spines within images were traced by using Neurolucida software (MicroBrightField Bioscience, Williston, VT) and analyzed using NeuroExplorer (MicroBrightField). Data represent the mean ± SEM (n = 16 neurons from four WT mice and n = 16 neurons from four ARHGAP10 KI/NHEJ male mice).

**Behavioral analysis**

Behavioral analysis of ARHGAP10 KI/NHEJ mice was performed at the age of 10 weeks old. We divided these mice into three groups A [WT male (n = 4), WT female (n = 5), ARHGAP10 KI/NHEJ male (n = 6) and ARHGAP10 KI/NHEJ female (n = 6)] and B [WT male (n = 4), WT female (n = 5), ARHGAP10 KI/NHEJ male (n = 5) and ARHGAP10 KI/NHEJ female (n = 6)] and C [WT male (n = 10), WT female (n = 10), ARHGAP10 KI/NHEJ male (n = 10) and ARHGAP10 KI/NHEJ female (n = 9)]. Behavioral tests were carried out following order; group A: open field test, Y-maze test, elevated plus maze test, locomotor activity test, novel object recognition test, social interaction test, prepulse inhibition test, fear conditioning test, light dark transition test. Group B: open field test, Y-maze test, elevated plus maze test, locomotor activity test, novel object recognition test, social interaction test, prepulse inhibition test, fear conditioning test, rotarod test and METH-induced hyperlocomotion test. In METH-induced hyperlocomotion test, we added the mice [WT male (n = 4) and ARHGAP10 KI/NHEJ male (n = 4)] to group B. Group C: open field test, Y-maze test, elevated plus maze test, locomotor activity test, novel object recognition test, social interaction test, prepulse inhibition test, fear conditioning test, rotarod test, METH-induced hyperlocomotion test and light dark transition test.

Open ﬁeld test was carried out as described previously.^14^ Mice were placed in the center of the arena and allowed to explore the open ﬁeld (diameter: 60 cm, height: 35 cm) for the following 5 min under moderately light conditions (85 lx). The open ﬁeld was divided into an inner zone (diameter: 40 cm), and an outer zone surrounding the inner zone. The movement of mice was measured via a camera mounted above the open ﬁeld and its activity was analyzed automatically using the Ethovision automated tracking program (Brainscience Idea Co., Ltd., Osaka, Japan). Measurements included distance and time spent in the inner and outer zone.

Y-maze test was carried out as described previously.^15^ Each arm is 40 cm long, 12 cm high, 3 cm wide at the bottom, and 10 cm wide at the top. The arms converge in an equilateral triangular central area that is 4 cm at its longest axis. Each mouse was placed individually at the central area and allowed to move freely through the maze during an 8 min session. The series of arm entries was recorded visually. Definition of alternative behavior was successive entries into the three arms, on overlapping triplet sets. The percent alternation is calculated as the ratio of actual to possible alternations (defined as the total number of arm entries minus 2) multiplied by 100. Spontaneous alternation was associated with the capacity of short-term memory.

The elevated plus maze was conducted as described previously with minor modifications.^16^ The apparatus was made of plastic material and was elevated to a height of 50 cm above the ground. Each arm of the plus maze was 16 cm in length and 10 cm in width. Additionally, the closed arms had wall enclosures that were 20 cm high. The central platform was a square of 10 × 10 cm. Light intensity around the maze was set at 100–120 lux. Mouse was placed on the elevated plus maze facing the open arm opposite to the experimenter. The number of entries and the time spent in the open and closed arms were recorded over the entire 5-min duration of the test.

Locomotor activity under a novel environment was conducted as described previously.^14^ Each mouse was placed in a standard transparent rectangular rodent cage (25 × 30 × 18 cm) under moderately light conditions (15 lx). Locomotor activity was then measured for 120 min using an infrared sensor (NS-DAS-8; Neuroscience, Tokyo, Japan) placed over the cage. To investigate the effect the sensitivity to methamphetamine hydrochloride (METH; Dainippon Sumitomo Pharma Co., Ltd., Osaka, Japan), each mouse was allowed a 120-min habituation period before METH (1 mg/kg, i.p.) treatment. Locomotor activity was then measured for 120 min immediately after METH treatment.

Novel object recognition test was carried out as described previously with minor modifications.^17^ Mice were individually habituated to an open-box (30 × 30 × 35 cm) for 3 days. During the training session, two novel objects were placed in the open ﬁeld and the animals were allowed to explore for 10 min under moderately light conditions (15 lx). The time spent exploring each object was recorded. During test sessions, one of the familiar objects used during training session was replaced by a novel object. The animals were placed back into the same box 24 h after the training session, and the mice were allowed to explore freely for 5 min. The preference index in the test session, the ratio of the amount of time spent exploring the novel object over the total time spent exploring both objects, was used to measure cognitive function. In the training session, the preference index was calculated as the ratio of time spent exploring the object that was replaced by a novel object in the test session, to the total exploration time.

The procedure for the social interaction and social novelty test were performed as previously described with minor modifications.^16^ The experiment was conducted under moderately light conditions (15 lx). The social interaction chamber is a three-chambered apparatus. Doorways built into the two dividing walls the mice can access to the each of the three chambers freely. Each chamber was 20 × 40.5 ×22 cm. In habituation session, each test mouse was individually habituated to chambers. In the sociability test, small plastic container confining an unfamiliar adult male (Stranger 1) was put one of the side chambers whereas empty small cage was placed in another side chamber. The duration of social interaction was recorded when the mouse spent within the 10 cm circle around plastic container. Subsequently the same experimental mouse was subjected to the test for social novelty, the second unfamiliar mouse being a new stranger mouse (Stranger 2) placed in the opposite side, which was previously empty during the sociability test. During social novelty test, the experimental mouse was allowed to explore all the three chambers for 10 min. The duration of social interaction with Stranger 1 or Stranger 2 was recorded in the same way as before. All stranger mice used in the experiment were WT mice. The entire procedures were measured automatically using the Ethovision automated tracking program (Brainscience Idea Co., Ltd., Osaka, Japan).

Prepulse inhibition (PPI) test was carried out as described previously.^14^ After the animals were placed in the chamber under moderately bright light conditions (180 lx) (San Diego Instruments, San Diego, CA), they were allowed to habituate for 10 min, during which 65 dB background white noise was present. The animals then received 10 startle trials, 10 no-stimulus trials and 40 PPI trials. The inter-trial interval was between 10 and 20 s and the total session lasted 17 min. The startle trial consisted of a single 120 dB white noise burst lasting 40 ms. PPI trials consisted of a prepulse (20 ms burst of white noise at 69, 73, 77 or 81 dB intensity) followed, 100 ms later, by the startle stimulus (120 dB, 40 ms white noise). Each of the four prepulse trials (69, 73, 77 or 81 dB) was presented 10 times. Sixty different trials were presented pseudo-randomly, ensuring that each trial was presented 10 times and that no two consecutive trials were identical. The resulting movement of the animal in the startle chamber was measured for 100 ms after startle stimulus onset (sampling frequency 1 kHz), rectiﬁed, ampliﬁed and fed into a computer, which calculated the maximal response over the 100-ms period. Basal startle amplitude was determined as the mean amplitude of the 10 startle trials. PPI was calculated according to the formula: 100 × [1 - (PPx/P120)]%, in which PPx was the mean of the 10 PPI trials (PP69, PP73, PP75 or PP80) and P120 was the basal startle amplitude.

Fear conditioning test was carried out as described previously.^15^ In the conditioning phase, each mouse is placed in the training cage (30 × 30 × 40 cm) equipped with a metal floor, and a 15-sec tone (85 dB) is delivered (conditioned stimulus). During the last 5 sec of the tone stimulus, a foot shock of 0.8 mA is delivered as an unconditioned stimulus through a shock generator. This procedure is repeated four times with 15-sec intervals. 24 hr after the conditioning, context-dependent test was carried out. For context-dependent test, mouse is placed in the training cage, and the freezing response is measured for 2 min in the absence of the conditioned stimulus. 4 hr after context-dependent test, tone-dependent test was carried out. For tone-dependent test, the freezing response was measured in the neutral cage for 1 min in the presence of a continuous-tone stimulus identical to the conditioned stimulus using mice which had been subjected to context-dependent test.

Light dark transition test was conducted as previously described with minor modifications.^18^ The apparatus used for the light/dark transition test consisted of a cage (15 × 15 × 15 cm) divided into two sections of equal size by a partition containing a door. One chamber was brightly illuminated (300 lux) whereas the other chamber was darker than 10 lux. Mice were placed into the dark side and allowed to move freely between the two chambers with the door open for 10 min. The time spent in each side and activity were recorded by using a MED-PC IV (Med Associations, Inc., St Albans City, USA).

Rotarod test was performed according to a previous report with minor modifications.^19^ In brief, Rotarod test was performed using the MK-600 (MUROMACHI KIKAI CO., LTD, Tokyo, Japan), under moderately light conditions (15 lx). The mice were trained for 3 days. During the training session, the mice were placed on a rod rotating at 6 revolutions per minute (rpm) and the time taken for them to fall from the rod was measured. If a mouse stayed on the rod until the end of the 2-min trial, a time of 120 s was recorded. Test session was carried out on day 4. The mice were placed on a rod rotating at 12 rpm and the time taken for them to fall from the rod was measured. Each mouse was subjected to 6 trials per day with a 15 min intertrial interval in the training and test session. We recorded the score the average value in a set of measurements. The apparatus was routinely cleaned with water and ethanol following each session.

**Statistical analysis in behavioral analysis**

Biological data are expressed as the mean ± SE. Differences between two groups were analyzed by two-tailed Student’s t-test. Differences in locomotor activity, PPI test, fear conditioning test and Rotarod test were analyzed by repeated analysis of variance (ANOVA). Multiple group comparisons were made by one-way ANOVA, followed by Tukey test when F ratios were signiﬁcant (p < 0.05).

**Refferences in supplementary materials**

1. Kato K, *et al.* The inositol 5-phosphatase SHIP2 is an effector of RhoA and is involved in cell polarity and migration. *Mol Biol Cell* 2012; **23**(13)**:** 2593-2604.

2. Garcia-Mata R, *et al.* Analysis of activated GAPs and GEFs in cell lysates. *Methods Enzymol* 2006; **406:** 425-437.

3. Yoshimi A, *et al.* Author Correction: Proteomic analysis of lymphoblastoid cell lines from schizophrenic patients. *Transl Psychiatry* 2019; **9**(1)**:** 146.

4. Livak KJ, Schmittgen TD. Analysis of relative gene expression data using real-time quantitative PCR and the 2(-Delta Delta C(T)) Method. *Methods* 2001; **25**(4)**:** 402-408.

5. Ran FA, *et al.* Genome engineering using the CRISPR-Cas9 system. *Nat Protoc* 2013; **8**(11)**:** 2281-2308.

6. Cong L, *et al.* Multiplex genome engineering using CRISPR/Cas systems. *Science* 2013; **339**(6121)**:** 819-823.

7. Vouillot L, Thelie A, Pollet N. Comparison of T7E1 and surveyor mismatch cleavage assays to detect mutations triggered by engineered nucleases. *G3 (Bethesda)* 2015; **5**(3)**:** 407-415.

8. Aida T, *et al.* Cloning-free CRISPR/Cas system facilitates functional cassette knock-in in mice. *Genome Biol* 2015; **16:** 87.

9. Sakuma T, *et al.* Repeating pattern of non-RVD variations in DNA-binding modules enhances TALEN activity. *Sci Rep* 2013; **3:** 3379.

10. Sakuma T, *et al.* Efficient TALEN construction and evaluation methods for human cell and animal applications. *Genes Cells* 2013; **18**(4)**:** 315-326.

11. Takuma K, *et al.* Ginkgo biloba extract EGb 761 attenuates hippocampal neuronal loss and cognitive dysfunction resulting from chronic restraint stress in ovariectomized rats. *Neuroscience* 2007; **149**(2)**:** 256-262.

12. Kuroda K, *et al.* Behavioral alterations associated with targeted disruption of exons 2 and 3 of the Disc1 gene in the mouse. *Hum Mol Genet* 2011; **20**(23)**:** 4666-4683.

13. Sobue A, *et al.* Genetic and animal model analyses reveal the pathogenic role of a novel deletion of RELN in schizophrenia. *Sci Rep* 2018; **8**(1)**:** 13046.

14. Ibi D, *et al.* Neonatal polyI:C treatment in mice results in schizophrenia-like behavioral and neurochemical abnormalities in adulthood. *Neurosci Res* 2009; **64**(3)**:** 297-305.

15. Ibi D, *et al.* Combined effect of neonatal immune activation and mutant DISC1 on phenotypic changes in adulthood. *Behav Brain Res* 2010; **206**(1)**:** 32-37.

16. Alkam T, *et al.* Evaluation of emotional behaviors in young offspring of C57BL/6J mice after gestational and/or perinatal exposure to nicotine in six different time-windows. *Behav Brain Res* 2013; **239:** 80-89.

17. Nagai T, *et al.* Dopamine D1 receptors regulate protein synthesis-dependent long-term recognition memory via extracellular signal-regulated kinase 1/2 in the prefrontal cortex. *Learn Mem* 2007; **14**(3)**:** 117-125.

18. Matsuo N, *et al.* Behavioral profiles of three C57BL/6 substrains. *Front Behav Neurosci* 2010; **4:** 29.

19. Okun E, *et al.* Evidence for a developmental role for TLR4 in learning and memory. *PLoS One* 2012; **7**(10)**:** e47522.

**Supplementary figure legends:**

**Supplementary Figure 1. Exonic CNVs of *ARHGAP10***

(a) Demographic data of the two sample sets in this study. (b) Six exonic deletions and one exonic duplication of *ARHGAP10* were validated by TaqMan copy number assays. Bars indicate copy number predicted by TaqMan copy number assays. CONT was used as a calibrator in each experiment. (c) Breakpoint sequencing for exonic duplication. Breakpoint sequencing revealed that the exonic duplication in Case #6 was tandem in direct orientation adjacent to the original locus. A black box in the electropherogram indicates overlapping sequences (TT) in the breakpoint. (d) Result of resequencing analysis for ARHGAP10. (e,f) Gene expression results of LCL. (e) Each dot [diamond: sample with deletion (Del), square: schizophrenia (SCZ), and triangle: controls (CONT)] represents the relative expression value of each sample calculated by the 2^-ΔΔCT^ method. The gray dots represent outlier values. (f) Results for statistical tests. Abbreviations: CONT, control; Del, deletion; SCZ, schizophrenia.

Abbreviations: CONT, Control

**Supplementary Figure 2. Generation of *Arhgap10-FLAG* knock-in mouse**

(a) Strategy of *Arhgap10-FLAG* reporter mice by CRISPR/Cas9 method. The guide RNA for targeted Cas9 cleavage was identical to *Arhgap10-3pV5-mCherry* in Supplementary Figure 4. Donor ssDNA coding FLAG tag was coinjected with Cas9/crRNA/tracrRNA mixture into nucleus of fertilized eggs. (b) PCR genotyping of *Arhgap10-FLAG* reporter mice. PCR primers for genotyping are shown in Supplementary Figure 3e. (c,d) Immunoblot analysis for detection of ARHGAP10 protein with FLAG tag using antibodies against (c) FLAG or (d) ARHGAP10 in brains from P28 *Arhgap10-FLAG* reporter mice. Predicted size of ARHGAP10-FLAG protein was 90 kDa. Brains were lysed in ice-cold buffer containing 20 mM HEPES-NaOH pH 7.5, 150 mM NaCl, 1% Triton-X100, 5 mM MgCl_2_, 1 mM DTT, and a protease inhibitor cocktail (Roche). Then the lysates were immunoprecipited with anti-FLAG (M2, Sigma Aldrich). (e) PCR primers for genotyping in this study.

**Supplementary Figure 3. *Arhgap10-3pV5-mCherry* knockin mice**

(a) The strategy of generating *Arhgap10-3pV5-mCherry* reporter knock-in mice by the CRISPR/Cas9 method. The guide RNA for targeted Cas9 cleavage was experimentally optimized on exon 23 (NP_084389.2) around the coding sequence junction and the 3'-UTR region. Donor dsDNA coding the *3pV5-mCherry* gene cassette was co-injected with the Cas9/crRNA/tracrRNA mixture into the nucleus of a fertilized egg. (b) PCR genotyping of *Arhgap10-3pV5-mCherry* reporter mice. The PCR primers for genotyping are shown in Supplementary Figure 3e. (c) For generation of *Arhgap10-3pV5-mCherry* reporter mice, we synthesized artificial genes encoding three tandem V5 tags and mCherry fluorescent protein (Eurofin, Japan). Both ends of this casette connect homologous regions of 300 bp to the 5' side and 80 bp to the 3' side, respectively. (d) Immunoblot analysis for V5 detection in various organs from postnatal day 28 (P28) *Arhgap10-3pV5-mCherry* reporter mice. The predicted size of the ARHGAP10-3pV5-mCherry protein was 120 kDa. The GAPDH signal was used as an internal control. (e, f) *Arhgap10-3pV5-mCherry* reporter knock-in mouse brain lysate was concentrated with V5-tag antibody conjugated agarose beads. The pull-down products, including the V5-tagged ARHGAP10 protein, were analyzed and confirmed by immunoblot using the (e) V5 antibody or (f) ARHGAP10 antibody. GAPDH was used as an internal control and could not be detected in pull-down lysates. (g-k) Immunoblot for detecting ARHGAP10-3pV5-mCherry protein in brain lysate. (g) Immunoblot analysis for V5 detection in various organs from P28 *Arhgap10-3pV5-mCherry* reporter mice. Predicted size of ARHGAP10-3pV5-mCherry protein is 120 kDa. GAPDH signal was used for internal control. Since the signals of the reporter protein were dependent of copy number of knockin allele, the expression of this reporter mice was proved to be specific enough. (h) Immunoblot analysis for V5 detection on embryonic day 15 (E15), P0 or P28 whole brain lysates. The asterisk indicates an unknown short isoform specific for developmental stage. (i) Immunocytochemistry of primary neurites, prepared from *Arhgap10-3pV5-mCherry* reporter mice, for co-expressions of Tuj1, V5-mCherry and tyrosine hydroxylase (TH). (j,k) Immonoblot analysis for V5 detection in DIV+3 and DIV+14 culture neurones. Relative intensities of the expression level of the *Arfgap10-3pV5-mCherry* reporter. TH or GAPDH were used as internal controls.

**Supplementary Figure 4. Generating model mice mimicking Case#5 (*Arhgap10* S490P/NHEJ).**

(a) Exon 17 of the *Arhgap10* gene was targeted by TALEN (NM_030113). Sequences of S490P knock-in allele and the NHEJ allele of Case #5 model mice. The green letters indicate the target p. S490P substitution. Ten base pairs were deleted in the NHEJ allele, resulting in a frameshift mutation. PCR primers for genotyping are shown in Supplementary Figure 2e. (b) Statistical analysis of neurite elongation and branch number in Case #5 model mouse. Primary cultured neuron from wildtype and S490P/NHEJ mutant embryos were plated and time-lapsed every 30 minutes for 3 days. Statistical processing of multiple t-test was performed using Prism 8 software. See also Figure 2. (c) Comparison of behavioral changes between WT and ARHGAP10 S490P/NHEJ (Case #5 model) in each test.

**Supplementary Figure 5. Behavior analysis of Case#5 model mouse**

Performance of *Arhgap10* S490P/NHEJ mice in open field test (a), locomotor test (b), novel object recognition test (c,d) and Y-maze test (e,f), light and dark box test (g,h), fear conditioning test (i,k), social interaction test (l-n), rotarod test (o,p), METH-induced hyperlocomotion test (2 mg/kg) (q,r). Data are represented as mean ± SEM (WT male (n = 18), WT female (n = 20), *Arhgap10* S490P/NHEJ male (n = 21) and *Arhgap10* S490P/NHEJ female (n = 21) in Fig S6a-d, i-n, q and r; WT male (n = 16), WT female (n = 20), *Arhgap10* S490P/NHEJ male (n = 20) and *Arhgap10* S490P/NHEJ female (n = 20) in Fig S6e, f; WT male (n = 14), WT female (n = 15), *Arhgap10* S490P/NHEJ male (n = 16) and *Arhgap10* S490P/NHEJ female (n = 15) in Fig S6g, H); WT male (n = 14), WT female (n = 15), *Arhgap10* S490P/NHEJ male (n = 15) and *Arhgap10* S490P/NHEJ female (n = 15) in Fig S6o, p; WT male (n = 12), WT female (n = 12), *Arhgap10* S490P/NHEJ male (n = 12) and *Arhgap10* S490P/NHEJ female (n = 11) in Fig S6s).

**Supplementary Figure 6. Histological analysis of Case#5 ARHGAP10 model mouse**

Nissl-stained brain sections of WT and *Arhgap10* S490P/NHEJ showed no deﬁcit in the development of the each region. (A: WT, B: *Arhgap10* S490P/NHEJ). Nissl-stained brain sections of WT (C-1, C-2) and *Arhgap10* S490P/NHEJ (D-1, D-2) showed no deﬁcit in the development of the medial prefrontal cortex.

Immuno-stained brain sections of WT (E-G) and *Arhgap10* S490P/NHEJ (H-J) showed no deﬁcit in the glia cells and neurons of the medial prefrontal cortex, which was stained with anti-GFAP (E and H), anti-Iba1 (F and I) and anti-NeuN (G and J).

Nissl-stained brain sections of WT (K-1, K-2) and *Arhgap10* S490P/NHEJ (L-1, L-2) showed no deﬁcit in the development of the hippocampus.

Immuno-stained brain sections of WT (M-O) and *Arhgap10* S490P/NHEJ (P-R) showed no deﬁcit in the glia cells and neurons of the hippocampus, which was stained with anti-GFAP (M and P), anti-Iba1 (N and Q) and anti-NeuN (O and R).

Nissl-stained brain sections of WT (S-1, S-2) and *Arhgap10* S490P/NHEJ (T-1, T-2) showed no deﬁcit in the development of the striatum.

Immuno-stained brain sections of WT (U-W) and *Arhgap10* S490P/NHEJ (X-Z) showed no deﬁcit in the glia cells and neurons of the striatum, which was stained with anti-GFAP (U and X), anti-Iba1 (V and Y) and anti-NeuN (W and Z).

**Supplementary Figure 7. Generated iPSCs can differentiate into three germ layers *in vitro***

(a) Evaluation of the capacity to differentiate into all three germ layers by SOX17 staining (endodermal marker), αSMA staining (mesodermal marker), and βIII-tubulin (ectodermal marker). Bars represent 100 μm. (b) Statistical analysis of neurite elongation from Case #5 iPS cells. Two clones from the control and two clones derived from case #5 were time-lapsed and measured every 12 hours for 60 hours. For the two clones of Case #5, the neurite length was compared at each time when Y-27528 was not added and when 1 μM or 10μM was added. Statistical processing of multiple t-test was performed using Prism8 software.
